# Design of Crosslinked Hydrogels Comprising Poly(Vinylphosphonic Acid) and Bis[2-(Methacryloyloxy)Ethyl] Phosphate as an Efficient Adsorbent for Wastewater Dye Removal

**DOI:** 10.3390/nano10010131

**Published:** 2020-01-10

**Authors:** Ismail Anil, Seyda Tugba Gunday, Ayhan Bozkurt, Omar Alagha

**Affiliations:** 1 Environmental Engineering Department, College of Engineering A13, Imam Abdulrahman Bin Faisal University, Main Campus, P.O. Box 1982, Dammam 34212, Saudi Arabia; oaga@iau.edu.sa; 2 Department of Biophysics, Institute for Research and Medical Consultations (IRMC), Imam Abdulrahman Bin Faisal University, Main Campus, P.O. Box 1982, Dammam 34212, Saudi Arabia; stgunday@iau.edu.sa (S.T.G.); abozkurt@iau.edu.sa (A.B.)

**Keywords:** poly(vinylphosphonic acid), methylene blue adsorption, wastewater dye removal, adsorption kinetic, adsorption isotherm

## Abstract

The development of adsorbents with high adsorption capacity and fast separation is of utmost importance for the environmental management of dye-bearing wastewaters. Within this scope, crosslinked hydrogels including poly(vinylphosphonic acid) (PVPA) and bis[2-(methacryloyloxy)ethyl] phosphate (BMEP) were designed with varying mole ratios of BMEP (5–40%). The Fourier transform infrared (FT-IR) spectroscopy, thermogravimetric analysis (TGA), scanning electron microscopy (SEM), transmission electron microscopy (TEM), and Brunauer-Emmett-Teller (BET) results revealed that the fabrication of crosslinked PVPA-BMEP hydrogels enhanced: (i) functionalities of PA groups in the structure of hydrogels, (ii) thermal stabilities up to 250 °C, and (iii) interaction between methylene blue (MB) molecules and hydrogels. The pseudo second-order kinetic model best described the experimental adsorption data. The behaviors of the isotherms were more appropriate for Langmuir than Freundlich isotherm for the experimental data. PVPA-BMEP (40%) hydrogel indicated a fast and an outstanding MB adsorption capacity of 2841 mg g^−1^, which has not been reported yet for polymer hydrogels, to the best of our knowledge. The thermodynamic studies concluded that MB adsorption process was spontaneous and exothermic in nature. The overall results suggest that the designed and fabricated PVPA-BMEP hydrogels have great potential for the efficient removal of coloring materials from wastewaters.

## 1. Introduction

The discharge of dye-bearing wastewaters arising from various industries such as textile, paper, printing, and leather into surface waters and groundwater is one of the most important environmental concerns due to the harmful effects on public health and the aquatic environment [1,2]. The dissolution of dyes in natural waters weakens the penetration of sunlight through water, and accordingly, photosynthetic capacities of aquatic plants decrease [3,4]. In addition to this, most of the synthetic dyes introduce their toxic, carcinogenic, and mutagenic properties into the food chain once they enter receiving water bodies as a result of their uncontrolled discharge or insufficient treatment [5,6].

The treatment process of synthetic dyes is a challenging task because of their complex, inert, non-biodegradable, and toxic structures [4,7]. Several methods, including adsorption, ion exchange, chemical coagulation, flotation, chemical oxidation, membrane filtration, reverse osmosis, ozonation, and photochemical degradation, have been used to treat synthetic dyes in wastewaters [1,8,9,10,11,12,13,14,15]. Adsorption has been proven to be a promising dye treatment process due to its substantial efficiency, simplicity, low cost, fast adsorbate/adsorbent contact time, etc. [1,8,9,16]. The removal capacity of the adsorption method may reach be up to 99.9%. The United States Environmental Protection Agency (USEPA) classified adsorption as one of the best wastewater treatment methods [17]. Disadvantages of the adsorption process are the regeneration capacity and regeneration cost of the adsorbent, biodegradability and disposal of the end-of-life adsorbent [18].

The ultimate fate of dyes, persistent pollutants in wastewater, is the marine environment. Consequently, they enter the food chain and reach the human at the top of the chain. The toxicity levels of cationic dyes are more than those of anionic dyes since they rapidly and easily interact with the surfaces of cell membranes, which are negatively charged [19]. Methylene blue (MB), one of the commonly used cationic dyes, is a significant basic dye with an aromatic structure. It has been widely used in medical, pharmaceutical, textile, chemical, aquaculture, and paper industries by virtue of its versatility. The growing demand for MB from these industries is expected to boost its supply in the market. Eye burns, skin irritation, irregular breathing, mental confusion, nausea, vomiting, and methemoglobinemia have been reported to be adverse health effects of MB unless its discharge to the environmental compartments is properly managed [20].

The academic and industrial domains have paid much attention to designing and manufacturing novel eco-friendly adsorbent materials with high adsorption capacity at a low dosage and fast adsorption kinetics in order to treat large volumes of MB-bearing wastewaters. The removal of MB from wastewater via an adsorption process has been investigated in numerous studies using various adsorbents such as activated carbon [2,21], clays [3,22], siliceous materials [23,24,25], bio-adsorbents [26,27], waste materials [28], nanoparticles [29,30], nanoadsorbents [31], and polymer hydrogels [32,33,34,35,36,37,38]. Polymer hydrogels have been found to be superior to other adsorbent materials mentioned here for the adsorption of MB in terms of outstanding adsorption capacities of over 2000 mg g^−1^.

There has been a remarkable effort to develop new polymer hydrogels with efficient dye adsorption characteristics. Among various functional polymer hydrogels, the interest in phosphorus-bearing polymers functionalized either at the side chain or at the main chain has recently been increasing due to their wide range of applications. Poly(vinylphosphonic acid) (PVPA) has solely represented the phosphorus-containing polymers in the commercial market so far [39]. The synthesis of PVPA from vinylphosphonic acid (VPA) is usually practiced by two common methods: (i) free-radical polymerization of VPA and (ii) VPA acid alkyl esters polymerization. The rigidity of the PVPA can be modified by adjusting the fraction of the phosphonic acid groups [40]. The PVPA indicates challenging properties because of the presence of phosphonic acid functional groups at every repeating unit in its structure. The PVPA-based products have been used in many applications such as polymer electrolyte membranes in fuel cells [41,42,43,44], drug delivery [45], dental cement [46], and corrosion and scale inhibition [47]. However, the existing literature indicates that very few studies have been performed on the dye adsorption capabilities of phosphonic acid-based hydrogels. Deka, et al. [48] developed highly phosphonic acid-functionalized organosilicas and tested their performances on removing different types of dyes. Their MB adsorption capacities reached 524 mg g^−1^ within 320 min. Sengel and Sahiner [49] designed PVPA nanogels with tailored properties and investigated their possible uses in biomedical and environmental applications. The MB adsorption capacity of the synthesized PVPA nanogels was found to be 14 mg g^−1^ at the end of 5 min of contact time. Nakhjiri, et al. [50] investigated the isotherm, kinetic, and thermodynamic parameters of MB adsorption by poly(acrylic acid-co-VPA) hydrogel cross-linked with N-maleyl chitosan, which resulted in 67 mg g^−1^ of adsorption capacity throughout 100 min. Herrera-Gonzalez, et al. [51] reported 417 mg g^−1^ of MB adsorption capacity within 360 min for the poly(VPA-co-triethylene glycol dimethacrylate) composite material that they developed.

In this study, PVPA and bis[2-(methacryloyloxy)ethyl] phosphate (BMEP)-bearing crosslinked hydrogels were designed and characterized. The used architectures containing phosphonic acid functional groups have the potential to enhance: (i) the flexibility and thermal stability of the hydrogels, (ii) the functionalities and pathways of the phosphonic acid groups in the structure of the hydrogels, and (iii) dye adsorption capacities of the hydrogels. The structural and morphological properties of PVPA hydrogels prepared with varying mole ratios of BMEP (5–40%) were characterized by Fourier transform infrared (FT-IR) spectroscopy, thermogravimetric analysis (TGA), and scanning electron microscopy (SEM) techniques for the first time. Thereafter, the removal of MB from an aqueous solution by adsorption onto PVPA-BMEP hydrogels was investigated through kinetic, isotherm, and thermodynamic models.

## 2. Materials and Methods

### 2.1. Chemicals

Vinylphosphonic acid 97% (VPA), Bis[2-(methacryloyloxy)ethyl] phosphate (BMEP), 2,2′-azobis(isobutyroic acid amidine)dihydrochloride (AIBA), methylene blue (C_16_H_18_ClN_3_S·3H_2_O), sulfuric acid (H_2_SO_4_, 95–97%), and sodium hydroxide (NaOH) pellets were purchased from Merck^©^ (KGaA, Darmstadt, Germany). All chemicals were used without further purification. The MB solutions required for batch adsorption experiments and calibration of spectrophotometer were prepared daily by dissolving the desired amount of MB in distilled water, which was produced by using a Milli-Q^©^ water purification system (Bedford, MA, USA).

### 2.2. Preparation of Poly(Vinylphosphonic Acid)-Bis[2-(methacryloyloxy)ethyl] Phosphate (PVPA-BMEP) Hydrogels

**Figure 1:**
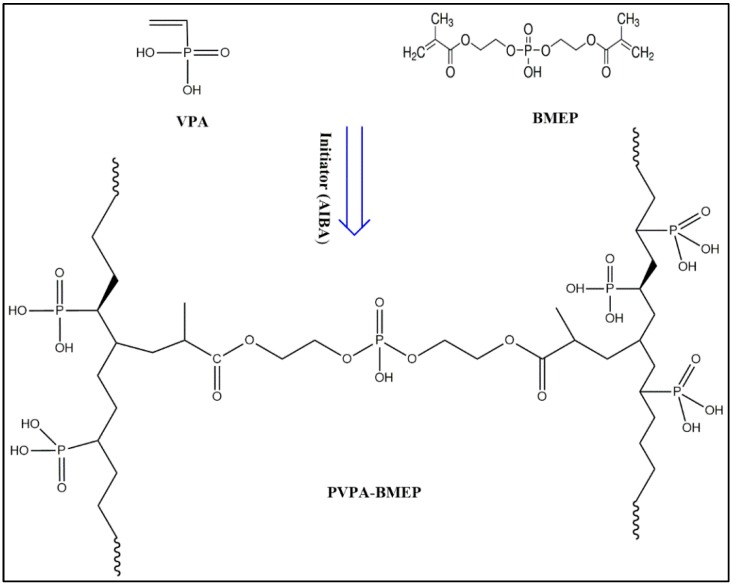
Schematic illustration for the synthesis of poly(vinylphosphonic acid)-bis[2-(methacryloyloxy)ethyl] phosphate (PVPA-BMEP) hydrogels.

The crosslinked PVPA-BMEP hydrogels were synthesized via free-radical polymerization of VPA in the presence of BMEP as a crosslinker and AIBA as an initiator (Figure 1). Several molar fractions of BMEP, from 5% to 40% with respect to VPA, were used in order to reveal the effect of BMEP on the flexibility, functionalities, and dye adsorption capacities of the crosslinked hydrogels. For instance, PVPA-BMEP (5%) was produced by using the following receipt: 1.0 g of VPA (9.3 mmole), 0.15 g of BMEP, 2.5 mg of AIBA (9.2 × 10^−3^ mmole), and 1.15 mL of distilled water were placed into a Schlenk flask that was evacuated and backfilled with Argon before the polymerization. The temperature of the reaction mixture was maintained at 80 °C for three hours. The solution was washed several times with ethanol to remove possible unreacted BMEPs. The insolubility and crosslinking of the PVPA with BMEP were tested by keeping the material in water. Then all the materials were dried under vacuum. The same preparation method was applied to synthesize the PVPA-BMEP (10%), PVPA-BMEP (20%), and PVPA-BMEP (40%) hydrogels by using 0.30 g, 0.59 g, and 1.19 g of BMEP during the synthesis, respectively. For the produced materials, high yields were obtained: 84% for PVPA-BMEP (5%), 88% for PVPA-BMEP (10%), 92% for PVPA-BMEP (20%), and 96% for PVPA-BMEP (40%).

### 2.3. Characterization of PVPA-BMEP Hydrogels

FT-IR spectra of hydrogels were measured in the range of 400–4000 cm^−1^ with a resolution of 4 cm^−1^ at room temperature by using a FT-IR spectrophotometer (Spectrum Two, PerkinElmer Inc., Massachusetts, USA). The TGA of the hydrogels were conducted by employing a simultaneous thermal analyzer (STA 6000, PerkinElmer Inc., Massachusetts, USA). The TGA data of hydrogels were acquired from 25 °C to 650 °C at a heating rate of 10 °C min^−1^ under N_2_ atmosphere with a flow rate of 20 mL min^−1^. The SEM (Inspect S50, FEI Company, Oregon, USA) was used to investigate the morphology of hydrogels. The hydrogels were coated with graphite prior to SEM imaging. The detailed surface morphology, structure analysis, and observation of the loading of MB molecules over hydrogels were performed by using transmission electron microscopy (TEM) (Morgagni 268, FEI Company, Brno, Czech Republic). Quantachrome Nova 2200e instrument (Anton Paar GmbH, Graz, Austria) was used for the determination of Brunauer-Emmett-Teller (BET) surface area, pore volume, and pore size of the hydrogels. The hydrogels were degassed at 100 °C for 6 h in N_2_. Multi-point BET measurements were done using N_2_ adsorption/desorption at 77 K. Zeta potential values of the hydrogels were determined by using a ZetaSizer instrument (Nano ZS, Malvern, UK). For zeta potential analysis, 20 mg of hydrogel was added into 10 mL solution with an ionic strength of 10 mM NaCl and varying pH values from 2 to 12. The final pH and zeta potential values were measured after each solution was suspended by stirring for 24 h.

### 2.4. Adsorption Studies

Batch adsorption experiments were performed in a set of 50 mL of amber glass Erlenmeyer flasks in order to minimize the possible photodegradation of MB molecules. The flasks containing different amounts of PVPA-BMEP hydrogels (5–100 mg) and 50 mL of MB solution with varying initial concentrations (100–1500 mg L^−1^) were stirred using digital magnetic stirrers with heating (Hei-Standard, Heidolph Instruments, Schwabach, Germany) at a stirring rate of 300 rpm and different temperatures of 25, 35, 45, and 55 °C for 180 min of contact time. The initial pH of the solution was adjusted using 1N of NaOH or 1N of H_2_SO_4_ solution. At pre-determined contact time intervals, aliquots were withdrawn, centrifuged at 5000 rpm for 5 min, and then the residual MB concentration in each solution was determined at 670 nm using a Hach-Lange DR 3900 spectrophotometer (Hach Company, Colorado, USA). The spectrometer was calibrated for each set of experiments by using six MB solutions with concentrations ranging from 0.5 to 50 mg L^−1^. The linear analytical curve was found to be [MB] (mg L^−1^) = 25.3 × Absorbance with R^2^ value of 0.999. The amount of MB adsorbed (mg) per gram of absorbent (q_t_) and MB removal efficiency of the absorbent at a specific contact time were calculated using Equations (1) and (2), respectively:

(1) $$q_{t}\;\left({\mathrm{mg}\;g}^{-1}\right)=\frac{\left(C_{0}\;-\;C_{t}\right)\;V}{M}$$

(2) $$\mathrm{RE}\;\left(\%\right)=\frac{C_{0}\;-\;C_{t}}{C_{0}}\;\times \;100$$

where C_0_ is the initial MB concentration and C_t_ is the MB concentration at a specific contact time (t) in the solution (mg L^−1^); V is the volume of the solution (L); and M is the mass of the PVPA-BMEP hydrogel in the solution (g).

### 2.5. Adsorption Kinetics

The adsorption process of dyes on adsorbents follows multi-stages; (i) surface reaction, (ii) diffusion through external and internal surfaces, and (iii) diffusion and interpenetration into the solid pores [52]. The kinetics and mechanisms of MB adsorption on PVPA-BMEP hydrogels were investigated using pseudo first-order (PFO) [53], pseudo second-order (PSO) [54], intraparticle diffusion (IPD) [55], and Elovich models [56]. The kinetic models, their linear forms in Equations (a)–(d), and the plot types to calculate the kinetic parameters of each model are shown in Table 1. In the Equations (a)–(d), q_e_ (mg g^−1^) is the amount of MB adsorbed at equilibrium time; k_1_ (min^−1^) is the equilibrium rate constant of the PFO model; k_2_ (g mg^−1^ min^−1^) is the equilibrium rate constant of the PSO model; k_d_ (mg g^−1^ min^−0.5^) is the constant rate and C (mg g^−1^) is the constant related to the resistance to diffusion of the IPD model; α (mg g^−1^ min^−1^) is the initial adsorption rate constant and β (g mg^−1^) is the desorption process constant of the Elovich model.

### 2.6. Adsorption Isotherms

The adsorption isotherm model study is necessary to elicit; (i) the interactions between an adsorbent and dye molecules, and (ii) the distribution of dye molecules in a liquid and solid phases until the equilibrium is reached [4,21]. The most widely applied isotherm models, including Langmuir [57], Freundlich [58], and Redlich–Peterson [59] were employed to assess the equilibrium data. The Langmuir model assumes that a monolayer adsorption process occurs on a homogeneous surface of an adsorbent while the Freundlich model depends on the assumption that an adsorption process on a surface of an adsorbent is governed by heterogeneous multilayer adsorption. The linear forms of Langmuir and Freundlich models are expressed in Equations (3) and (4), respectively:

(3) $$\frac{C_{e}}{q_{e}}=\frac{C_{e}}{q_{m}}+\frac{1}{q_{m}K_{L}}$$

(4) $$\ln\left(q_{e}\right)=\ln\left(K_{F}\right)+\frac{1}{n}\ln\left(C_{e}\right)$$

where C_e_ is the equilibrium concentration of MB in the solution (mg L^−1^), q_m_ (mg g^−1^) is the theoretical maximum adsorption capacity of the adsorbent, and K_L_ (L mg^−1^) is the constant of the Langmuir model; K_F_ (mg g^−1^) is the isotherm constant and 1/n is the dimensionless adsorption intensity of the Freundlich model.

The dimensionless constant regarding the favorableness of the adsorption, also known as the separation factor or equilibrium parameter (R_L_), can be determined using the Langmuir isotherm and represented in Equation (5):

(5) $$R_{L}=\frac{1}{1+K_{L}C_{o}}$$

The R_L_ value suggests that the adsorption process to be irreversible if R_L_ = 0, favorable if 0 < R_L_ < 1, linear if R_L_ = 1, and unfavorable if R_L_ > 1 [4].

The Redlich–Peterson isotherm, an empirical three-parameter model, is a combination of the elements from both Langmuir and Freundlich isotherms. Thus, this isotherm model is practicable for either homogenous or heterogeneous adsorption systems. The Redlich-Peterson isotherm model is defined by the following non-linear expression in Equation (6):

(6) $$q_{e}=\frac{K_{\mathrm{rp}}C_{e}}{1+\alpha_{\mathrm{rp}}C_{e}^{\beta }}$$

where K_rp_ (L g^−1^) and α_rp_ (L mg^−1^) are the Redlich-Peterson constants and β is the dimensionless exponent ranging between 0 and 1. The above equation becomes the Langmuir isotherm if β = 1, while it is reduced to the Freundlich isotherm if β = 0.

The convenience of these three models to describe the kinetic data was confirmed by calculating the normalized standard deviation (Δq_e_ (%)) given in Equation (7):

(7) $$\Delta q_{e}=100\sqrt{\frac{\sum {\left[\left(q_{e,\exp}-q_{e,\mathrm{cal}}\right)/q_{e,\exp}\right]}^{2}}{n-1}}$$

where q_e,exp_ (mg g^−1^) is the experimental adsorption capacity of the hydrogels, q_e,cal_ (mg g^−1^) is the calculated adsorption capacity by the isotherm model, and n is the number of experiments.

### 2.7. Adsorption Thermodynamics

Throughout the adsorption process, the mechanism and feasibility of the adsorption process are dominated by the variations in the thermodynamic parameters including standard free Gibbs energy (ΔG°), the standard enthalpy change (ΔH°), and the standard entropy change (ΔS°). These thermodynamic parameters are computed using the Van’t Hoff equations Equations (8) and (9) [60]:

(8) $$\ln\left(K_{D}\right)=-\frac{\Delta H{}^{\circ}}{R}\frac{1}{T}\;+\;\frac{\Delta S{}^{\circ}}{R}$$

(9) ΔG°=ΔH°−TΔS°

where K_D_ is the thermodynamic equilibrium constant which is calculated by plotting ln(q_e_/C_e_) against q_e_ where q_e_ is extrapolated to zero [7,61]; R is the universal gas constant (8.314 J K^−1^ moL^−1^); T is the absolute temperature. The thermodynamic parameters ΔH° and ΔS° are calculated by plotting ln(K_D_) against 1/T.

## 3. Results and Discussion

### 3.1. Characterization of PVPA-BMEP Hydrogels

#### 3.1.1. Fourier Transform Infrared (FT-IR) Spectra

**Figure 2:**
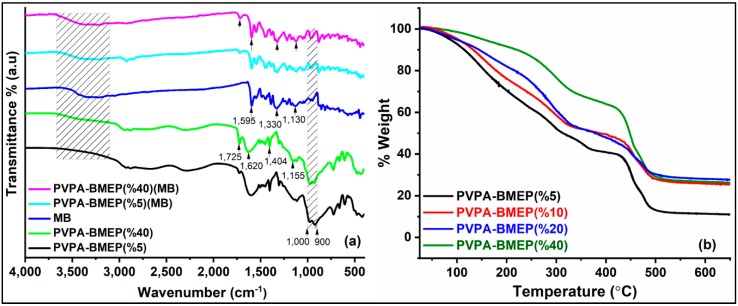
(**a**) Fourier transform infrared (FT-IR) spectra of methylene blue (MB) and PVPA-BMEP hydrogels with 5 and 40% of BMEP fractions before and after MB adsorption, (**b**) thermogravimetric (TG) spectra of PVPA-BMEP hydrogels.

The FT-IR spectra of MB and PVPA-BMEP hydrogels with 5 and 40% of BMEP fractions before and after MB adsorption are represented in Figure 2a. The characteristic broad peak appearing between 1000 and 900 cm^−1^ can be assigned to the (P-O)-H stretching of phosphonic acid groups of the PVPA. The peak at 1155 cm^−1^ is ascribed to the P-O stretching of BMEP and becomes more intense with increasing BMEP fraction in the matrix. The absorption band at 1404 cm^−1^ is assigned to the stretching vibration of C=O in BMEP. The phosphonic acid group gives additional broad band in the region of 1620 cm^−1^. The carboxyl group (C=O) stretching is clearly seen at 1725 cm^−1^, which becomes more intense as the fraction of BMEP in hydrogel increases [62]. The narrow and weak bands at 2880 cm^−1^ and 2950 cm^−1^ are due to the CH_2_ stretching vibrations in methyl and methylene groups of BMEP. The broadening peak within 3300–2000 cm^−1^ is attributed to hydrogen bonding network formation among phosphonic acid groups [63]. These results confirm that the crosslinking of the PVPA with BMEP was successfully performed to produce PVPA-BMEP hydrogels. After MB adsorption onto the hydrogels, the characteristic twin bands between 1000–900 cm^−1^ due to the stretching vibrations of the P-OH group were masked by MB peaks. The band at 1155 cm^−1^ (P-O) and the broad peak centered at 1620 cm^−1^ (phosphonic acid group) disappeared after MB adsorption [64]. It is also evident that the intensities of the peaks at 1404 cm^−1^ (C=O) and 1725 cm^−1^ (C=O) decreased after the adsorption [50]. The main distinction of MB FTIR spectra is the presence of a broad and intense band between the region 3100–3600 cm^−1^ due to the vibrations of OH groups bonded with nitrogen atoms of the MB heterocycle and nitrogen atoms of the unsaturated (CH_3_)_2_ group. In addition to this, MB shows major spectral absorptions at 1595 cm^−1^ (for C=C and C=N bonds), 1330 cm^−1^ (for N-(CH_3_)_2_), and 1130 cm^−1^ (for C–N bonds) [24,34,65]. The appearance of these distinct MB bands on hydrogels after the adsorption confirms the effective MB adsorption by hydrogels. The distinct MB peaks become more intense as the BMEP content in the hydrogels increases. This efficient interaction could be clarified by the electrostatic attraction between MB molecules and deprotonated phosphonic acid units. It is well known that phosphonic acid has capability to deprotonate to form P-O-ions which attract cationic MB molecules to form a complex throughout the hydrogel matrix.

#### 3.1.2. Thermogravimetric Analysis (TGA) Study

Figure 2b depicts the TGA curves of PVPA-BMEP hydrogels. The hydrogels indicate similar three-step weight loss pattern within the temperature range of measurement. In the first step, a slight weight loss is observed for all hydrogels up to 100 °C, which is attributed to the loss of absorbed humidity. Beyond this point, the weight loss rate of hydrogels becomes faster up to 320 °C due to the loss of free water and further condensation reactions among phosphonic acid groups in the polymer matrix. Then, this step is followed by the degradation of the whole cross-linked material above 450 °C. The TGA results conclude that (i) thermal stabilities of the hydrogels are enhanced by increasing the BMEP fraction and (ii) all hydrogels have promising thermal stabilities for the dye adsorption applications. Besides these, the thermal stability of PVPA-BMEP (40%) hydrogel has been found to be superior to others up to 250 °C.

#### 3.1.3. Scanning Electron Microscope (SEM) Images

**Figure 3:**
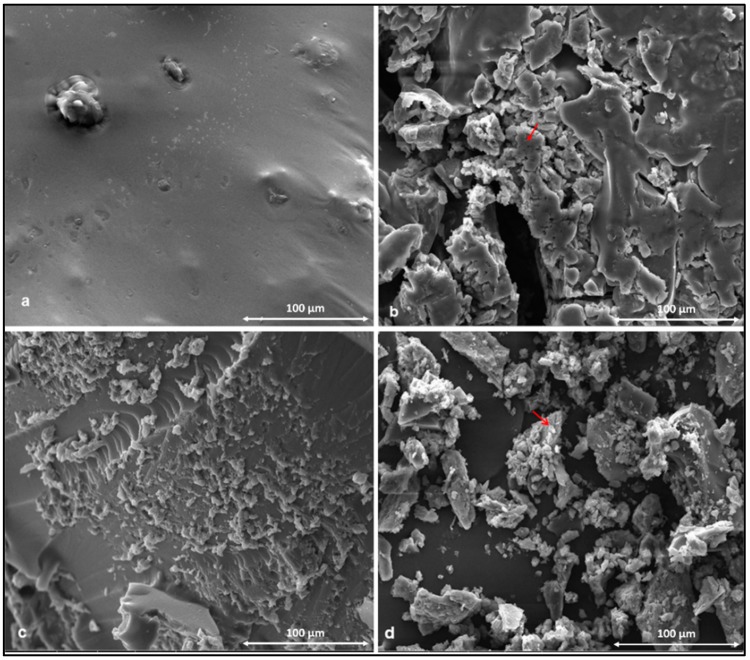
Scanning electron microscope (SEM) images of hydrogels: (**a**): PVPA-BMEP (5%), (**b**): PVPA-BMEP (5%) after MB adsorption, (**c**): PVPA-BMEP (40%), and (**d**): PVPA-BMEP (40%) after MB adsorption. Red arrows: MB molecules.

SEM images of PVPA-BMEP (5%) and PVPA-BMEP (40%) hydrogels before and after MB adsorption are illustrated in Figure 3. Figure 3a shows the SEM image of PVPA-BMEP (5%) hydrogel with a smooth surface morphology bearing bright domains on the crosslinked polymer. A similar structure can be observed for PVPA-BMEP (40%) hydrogel (Figure 3c). However, it is evident that the surface roughness of PVPA-BMEP (40%) is more than that of PVPA-BMEP (5%), which could be described by the existence of higher crosslinker fraction in PVPA-BMEP (40%) that affected the surface morphology.

After MB adsorption, the surface roughness of each hydrogel becomes apparent with certain porosity where the MB absorption is more feasible (Figure 3b,d). Additionally, the surface roughness and surface coverage by MB molecules (red arrows in Figure 3b,d) identified for the PVPA-BMEP (40%) hydrogel are considerably higher than those of PVPA-BMEP (5%). This result could lead to enhanced MB adsorption capacity for the hydrogels as the BMEP fraction increases.

#### 3.1.4. Transmission Electron Microscope (TEM) Images

**Figure 4:**
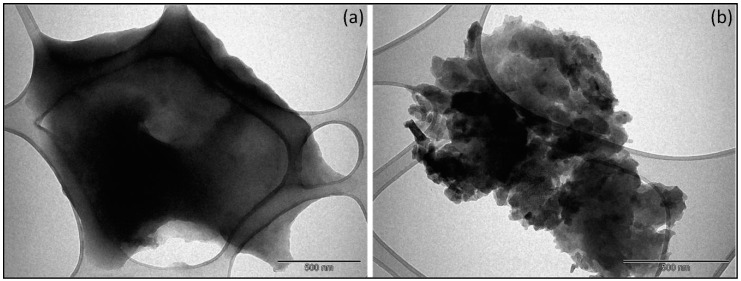
Transmission electron microscope (TEM) images of hydrogels (scale bar = 500 nm): (**a**): PVPA-BMEP (5%) and (**b**): PVPA-BMEP (40%).

TEM images of PVPA-BMEP (5%) and PVPA-BMEP (40%) hydrogels are illustrated in Figure 4. It is evident that there is not a significant change in the morphology of PVPA-BMEP (5%), which could be described by a homogeneous character of the final material with low porosity and mere aggregation. In the meantime, PVPA-BMEP (40%) hydrogel comprising the highest BMEP fraction exhibited spherical agglomerations and numerous cross-linked polymeric particles with a diameter ranging between 50–100 nm. This behavior could explain more efficient MB adsorption of PVPA-BMEP (40%) where the particles have bigger surface area to interact with more MB molecules. These findings are in a good agreement with the BET results where the surface area of the PVPA-BMEP (40%) hydrogel (10.2 m^2^ g^−1^) is bigger compared to that of PVPA-BMEP (5%) hydrogel (7.17 m^2^ g^−1^).

### 3.2. Effect of Adsorption Parameters

#### 3.2.1. Initial Solution pH

**Figure 5:**
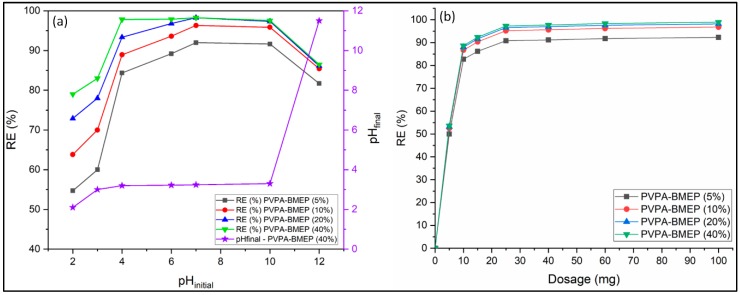
Effect of initial solution pH (**a**) and adsorbent dosage (**b**) on MB adsorption.

Solution pH is one of the most significant factors governing the dye adsorption process due to its substantial effects on the ionization and electrical charge of functional groups on the adsorbent surface [4,25,33,34]. The effect of the initial solution pH on the MB removal efficiencies of hydrogels was studied within the pH range between 2 and 12 (Figure 5a). Other parameters such as hydrogel mass, contact time, initial MB concentration, and temperature were kept constant at 25 mg, 180 min, 100 mg L^−1^, and 25 °C, respectively. Figure 5a indicates that MB removal capacities of hydrogels show similar behavior against the initial solution pH. The MB removal by hydrogels represents an important increasing pattern up to the pH value of 6, and then it remained almost constant with further pH increase up to 10. The MB removal efficiencies of hydrogels decreased about 11% when the initial solution pH = 12. The lowest MB removal rates of the PVPA-BMEP (5%), PVPA-BMEP (10%), PVPA-BMEP (20%), and PVPA-BMEP (40%) hydrogels were found to be 55, 64, 73, and 79%, respectively, at the initial solution pH value of 2. On the other hand, PVPA-BMEP (5%), PVPA-BMEP (10%), PVPA-BMEP (20%), and PVPA-BMEP (40%) hydrogels indicated their highest removal efficiencies as 92%, 96%, 97%, and 98%, respectively, at pH = 7.

In order to explain this behavior, the difference between the initial solution pH and the final solution pH after MB adsorption (ΔpH = pH_final_ − pH_initial_) was plotted against the initial solution pH (Supplementary Figure S1). The point where ΔpH = 0 was considered to be the pH at the point of zero charge (pH_PZC_) [21,61,66]. The electrical charge density of an adsorbent’s surface is assumed to be zero at pH_PZC_ value. Zeta potential values of PVPA-BMEP (40%) hydrogel were also depicted against the initial solution pH (Figure S1). The pH_PZC_ value of PVPA-BMEP (40%) hydrogel was found to be 3 by using both methods mentioned above. When the solution pH < pH_PZC_, the electrical charge of hydrogel becomes positive due to the protonation of phosphonic acid functional groups on the adsorbent surface. Thus, hydrogels start to repulse the cationic MB molecules. Meanwhile, MB molecules compete with the high concentration of hydrogen ions for the available adsorption sites on the hydrogels. These two mechanisms could explain the poor MB removal capacity of hydrogels at lower pH values. On the other hand, the surface of the hydrogels holds negatively charged binding sites because of the deprotonation of surface functional groups when the solution pH > pH_PZC_. Hereby, a strong electrostatic interaction between cationic MB molecules and anionic hydrogels could be ensured. For this reason, MB removal efficiencies of the hydrogels significantly enhanced up to pH 10. The further pH increase considerably weakened the MB removal due to the: (i) limited movement of MB molecules shielded by abundant OH^-^ to the hydrogels, and (ii) neutralization of cationic MB molecules with plenty of present OH^-^ in the solution [33,66,67,68].

Based on these results, pH value of 7 was determined as the optimal initial solution pH and, it was applied for further experiments. Most of the previous studies also reported the optimal pH value as 7 for MB adsorption by various types of hydrogels such as chitosan-g-poly (acrylic acid)/vermiculite composite [69], gum Ghatti and acrylic acid-based biodegradable hydrogels [70], sodium alginate-based organic/inorganic superabsorbent composites [71], poly(acrylic acid-co- vinylphosphonic acid) hydrogel cross-linked with N-maleyl chitosan [50], and natural clay mineral [3]. In conclusion, the results obtained from the effect of solution pH on MB removal studies revealed that the MB removal efficiencies of the hydrogels are significantly dependent on the solution pH. The physical adsorption via electrostatic interaction is probably the most important mechanism that controls the adsorption capacities of the hydrogels.

#### 3.2.2. Adsorbent Dose

The effect of adsorbent dose on the MB adsorption was studied by implementing various amounts of hydrogels ranging from 5 to 100 mg at an initial MB concentration of 250 mg L^−1^ and temperature of 25 °C for 3 h of contact time. It is evident from Figure 5b that the increase in doses from 5 to 25 mg significantly improves the MB removal efficiencies of all hydrogels. This could be explained by that the availability of the surface area and adsorption sites of the adsorbent and dye-adsorbent interactions are enhanced by increasing the adsorbent dose. On the other hand, the further increase in adsorbent dosage slightly improves the MB removal, suggesting the unsaturated adsorption sites through the adsorption process. The MB removal efficiencies of PVPA-BMEP (5%), PVPA-BMEP (10%), PVPA-BMEP (20%), and PVPA-BMEP (40%) against increasing dose indicate similar patterns and reach 90.8%, 95.2%, 96.6%, and 97.3%, and respectively at 25 mg of dosage. These results also point to the fact that increasing the BMEP fraction enhances MB removal efficiency most probably due to the increased number of phosphonic acid functional groups on the surface of the hydrogel. It is worth mentioning that increasing the adsorbent dose from 25 mg to 100 mg improves the MB removal efficiency only by 1.6%, which may not be a cost-effective and environmentally friendly approach. Therefore, 25 mg of adsorbent amount was selected as the optimal dose and implemented in further experiments.

#### 3.2.3. Contact Time

**Figure 6:**
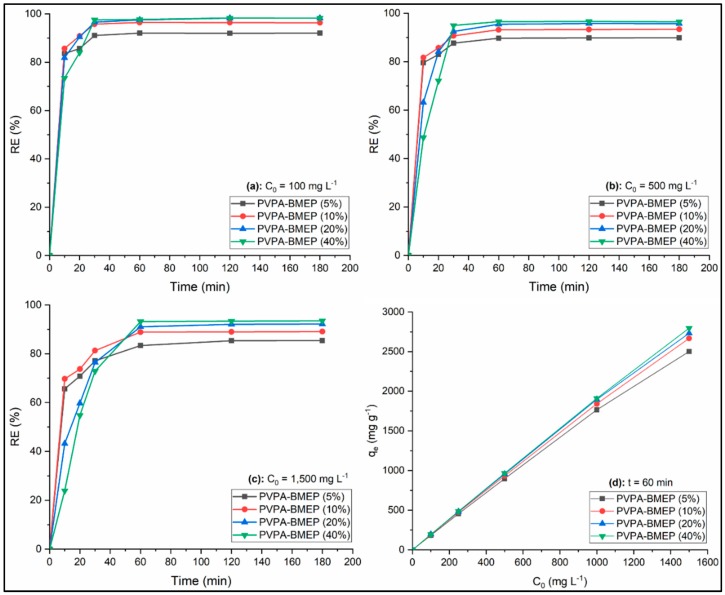
Effect of contact time (**a**–**c**) and initial MB concentration (**d**) on MB adsorption.

The MB removal efficiencies by PVPA-BMEP hydrogels at the contact times throughout 10 and 180 min at three levels of initial MB concentrations (100, 500, and 1500 mg L^−1^) were investigated and depicted in Figure 6a–c, respectively. The results for initial MB concentrations of 100 and 500 mg L^−1^ reveal that MB removal efficiencies of all hydrogels are quite fast within the first 30 min of contact time and do not significantly improve with further increase in contact time. On the other hand, the hydrogels reach the adsorption equilibrium at 60 min of contact time for initial MB concentration of 1500 mg L^−1^. Thus, the optimal contact time was chosen as 60 min for the following experiments. The instantaneous MB adsorption behaviors of the hydrogels could be attributed to the fast diffusion of MB molecules at high concertation gradient and available active adsorption sites of the hydrogels. Following this, the decrease in the number of adsorption sites of the hydrogels due to the saturation and MB concentration in the solution results in lower adsorption rates. It can also be concluded from Figure 6 that the hydrogels with lower BMEP fractions (5% and 10%) provide faster MB removal until the equilibrium time while MB adsorption performances of hydrogels at adsorption equilibrium improve as their BMEP fractions increase (20% and 40%). PVPA-BMEP (40%) hydrogel indicated the best MB removal efficiencies of 97.7%, 96.5%, and 93.2% for initial MB concentrations of 100, 500, and 1500 mg L^−1^, respectively.

#### 3.2.4. Initial Dye Concentration

Figure 6d shows the effect of initial MB concentration ranging from 100 to 1500 mg L^−1^ on the equilibrium adsorption capacities of the hydrogels. Obviously, the q_e_ value of each hydrogel linearly increases when the initial MB concentration is increased, suggesting that the initial MB concentration dominates the adsorption process. This could be linked to (i) higher driving force at elevated MB concentration to cope with the mass transfer resistance of MB [26,72]; (ii) enhanced interaction between MB molecules and hydrogels [28]; (iii) availability of more functional sites on surface of hydrogels and surface roughness, as confirmed from FT-IR and SEM characterization results. Besides these, the slopes of the curves for q_e_ against C_0_ (Figure 6d) start to gradually decrease when C_0_ value increases. In particular, the declines in the slopes become more notable as the BMEP fraction of the hydrogel decrease. These adsorption capacity tendencies of the hydrogels are supported by the results shown in Figure 6a–c where the MB removal efficiencies of hydrogels decrease as the C_0_ value increases. This could be a result of the initiation of the available binding sites on the hydrogel’s surface for saturation [73]. It was found that q_e_ values of PVPA-BMEP (20%) and PVPA-BMEP (40%) are higher than those of hydrogels with lower BMEP fractions and their q_e_ values improve from 195.1 to 2766 mg g^−1^ and from 195.4 and 2805 mg g^−1^, respectively, as C_0_ increases from 100 to 1500 mg L^−1^.

### 3.3. Adsorption Kinetics

**Figure 7:**
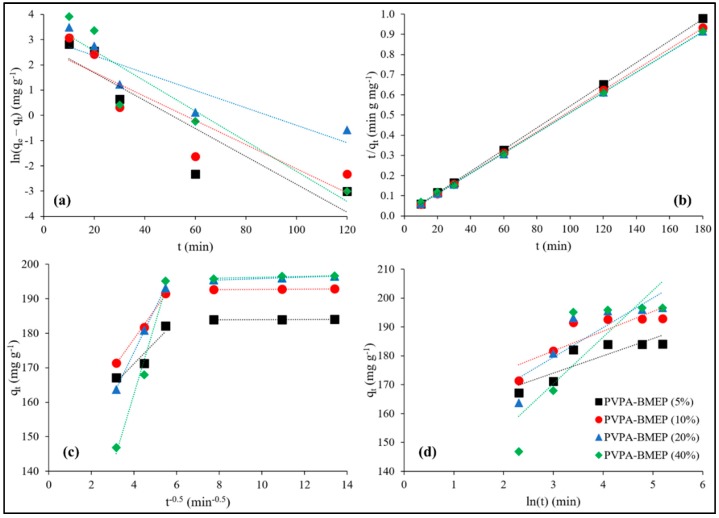
The linear fits of kinetic models to the experimental data at C_0_ = 100 mg L^−1^; (**a**): pseudo first-order (PFO), (**b**): pseudo second-order (PSO), (**c**): intraparticle diffusion (IPD), and (**d**): Elovich model.

Adsorption kinetic studies were thoroughly investigated at three levels of C_0_ (100, 500, and 1500 mg L^−1^), contact times from 10 to 180 min, and temperature of 25 °C. The kinetic parameters and the coefficient of determination (R^2^) values of PFO, PSO, IPD, and Elovich models were calculated by using the equations, and the plot types listed in Table 1 and the results are represented in Table 2 accordingly. The linear fits of kinetic models to the experimental data for C_0_ = 100 mg L^−1^ are shown in Figure 7. Table 2 reveals that, in all cases, the experimental data fit best to PSO kinetic model (R^2^_avg_ = 0.997 ± 0.005) then followed by PFO (R^2^_avg_ = 0.858 ± 0.066), Elovich (R^2^_avg_ = 0.792 ± 0.075), and IPD (R^2^_avg_ = 0.636 ± 0.091) kinetic models, respectively. In addition to this, the calculated q_e_ values of the PSO model are quite closer to the experimental q_e_ values than those of other kinetic models. These results estimated by using kinetic models might be an indication that MB adsorption onto hydrogels is possibly governed by chemisorption, comprising exchanging or sharing of electrons between the MB cations and surface phosphonic acid functional groups of the hydrogels [28,52,73].

The IPD model was further employed to examine the kinetic data in order to specify whether IPD is the rate-limiting step in the MB adsorption on the hydrogels. Figure 7c indicates multilinear plots of the IPD process of MB adsorption onto hydrogels, suggesting that the adsorption process follows two distinct stages. The k_d_, C, and R^2^ values of IPD model for these two stages of MB adsorption onto hydrogels could be found in Supplementary Table S1. The first linear region is ascribed to the external diffusion, where MB molecules quickly diffuse from solution to the exterior surface of hydrogels. The second linear region is commonly linked to the IPD stage if an adsorbent has a rough surface and available pores with active adsorption sites [21,61,74,75]. The slopes of the linear plots (k_d1_ values) of the first region are substantially higher than k_d2_ values of the second region for all hydrogels at each C_0_ level, explaining that surface adsorption is the dominating mechanism rather than the IPD. The highest k_d1_ values accompanied by lowest C_1_ values for the hydrogels with 20% and 40% BMEP fractions points out that these hydrogels have more adequate adsorption sites and surface area. Consequently, IPD is not the rate-limiting step since the values of C, C_1_, and C_2_, representing the intercept of the linear plot or the thickness of boundary layer, are not equal to zero for all hydrogels at each C_0_ level (Table 2 and Table S1).

The Elovich model’s principle assumes that the removal of adsorbate from a solution ensues from chemical adsorption on an energetically heterogeneous surface of an adsorbent [21,75]. It can be concluded from Table 2 and Figure 7d that the Elovich model is not able to predict the experimental adsorption data well since the R^2^ values varies between 0.699 (for PVPA-BMEP (20%) at C_0_ = 500 mg L^−1^) and 0.924 (for PVPA-BMEP (5%) at C_0_ = 1500 mg L^−1^) with an average R^2^ value of 0.792 ± 0.075 for the whole kinetic data. On the other hand, R^2^ values have an increasing trend as the C_0_ values increases, resulting in improvement in the model’s fitting performance. Moreover, a remarkable decline in both α and β values for all hydrogels resulting from the increase in the C_0_ values could be attributed to desorption and possibly represent irreversible MB adsorption onto hydrogels [21,75]. Hereby, the heterogeneous chemisorption character of the hydrogels at elevated MB concentrations could be noteworthy, although the entire kinetic dataset does not agree well with the Elovich model.

### 3.4. Adsorption Isotherms

**Figure 8:**
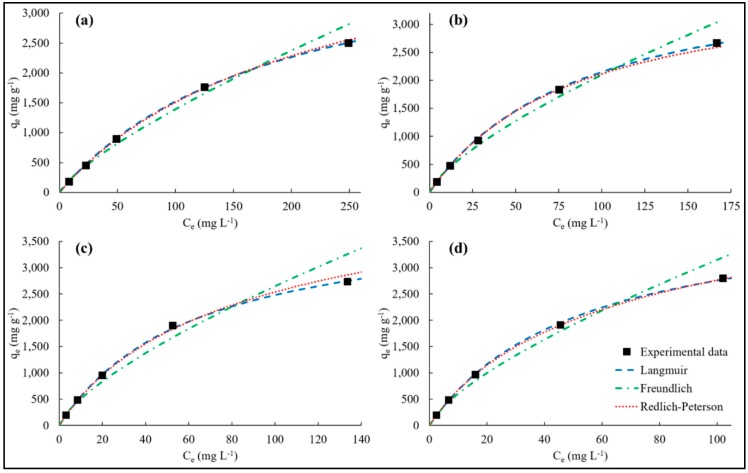
The adsorption isotherms of MB on (**a**): PVPA-BMEP (5%), (**b**): PVPA-BMEP (10%), (**c**): PVPA-BMEP (20%), and (**d**): PVPA-BMEP (40%).

The theoretical maximum adsorption capacity of the adsorbent (q_m_) and the constant of the Langmuir model (K_L_) were calculated from the plot C_e_/q_e_ against C_e_ while the isotherm constant (K_F_) and the dimensionless adsorption intensity (1/n) of the Freundlich model were calculated from the plot ln(q_e_) against ln(C_e_). The K_rp_, α_rp_, and β parameters of the Redlich–Peterson model were calculated by using the non-linear regression method of the Solver tool in Microsoft Excel software. Table 3 shows the calculated parameters of the isotherms and their respective R^2^ and Δq_e_ values. The adsorption isotherms of MB on the hydrogels at initial MB concentrations varying between 100 and 1500 mg L^−1^ are depicted in Figure 8.

The R^2^ values of Langmuir, Freundlich, and Redlich–Peterson isotherm models reveal that each model describes the experimental data well (R^2^ ≥ 0.981). However, the Langmuir and Redlich–Peterson isotherm models fit the experimental data better, considering the higher R^2^ (≥0.992) and lower Δq_e_ values (≤3.31%). The MB adsorption onto PVPA-BMEP (10%) and PVPA-BMEP (20%) hydrogels are described best by Langmuir model while Redlich–Peterson model explains the experimental data of the PVPA-BMEP (5%) and PVPA-BMEP (40%) hydrogels quite well, on the basis of the highest R^2^ and the lowest Δq_e_ values.

The Langmuir model results specify that increasing BMEP fraction in the hydrogels results in higher q_m_ values. The highest q_m_ and K_L_ values were calculated to be 2841 mg g^−1^ and 19.2 × 10^−3^ L mg^−1^, respectively, for the PVPA-BMEP (40%) hydrogel because of the enhanced functionalities and pathways of the phosphonic acidic groups in its structure. The calculated R_L_ values for hydrogels shown in Supplementary Figure S2 affirm that the MB adsorption process onto hydrogels is favorable because all R_L_ values are between 0 and 1. Additionally, the R_L_ values of the hydrogels follow logarithmic decrement pattern against increasing C_0_ values, implying that the adsorption is more favorable and also more irreversible since R_L_ values get closer to zero at higher C_0_ values. The lower R_L_ values found for the hydrogels with higher BMEP fractions (20% and 40%) over the whole C_0_ range further verify stronger interaction with MB molecules.

The dimensionless adsorption intensity of the Freundlich model, 1/n, can be used to reveal the favorability of an adsorption process and the surface heterogeneity of an adsorbent. The calculated 1/n values of all hydrogels varying between 0 and 1 imply that MB molecules are favorably adsorbed by the hydrogels. This is in good agreement with the results regarding the R_L_ values of the Langmuir model. The surface heterogeneity is expected to be greater when the value of 1/n gets close to zero. The surface heterogeneity of the hydrogels slightly increases with increasing BMEP fraction. The 1/n values of the PVPA-BMEP (5%), PVPA-BMEP (10%), PVPA-BMEP (20%), and PVPA-BMEP (40%) were found to be 0.769, 0.721, 0.716, and 0.710, respectively. The SEM image of the PVPA-BMEP (40%) (Figure 3c) corroborates the increased surface heterogeneity due to the highest number of functional phosphonic acid groups existing on the surface of this hydrogel.

The Redlich–Peterson model was applied to figure out a compromise between Langmuir and Freundlich systems. The β values of the hydrogels obtained by the model were close to unity, presenting that the behaviors of the isotherms are more appropriate for Langmuir than Freundlich isotherm. The results here acquired by using adsorption isotherm models propose that the MB adsorption process on the hydrogels’ surface is mainly dominated by the homogeneous monolayer adsorption with the partial contribution of heterogeneous adsorption.

### 3.5. Adsorption Thermodynamics

**Figure 9:**
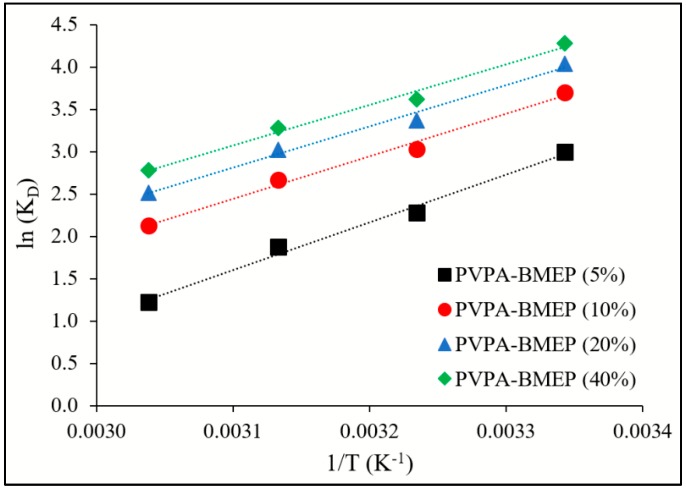
Linear plots of ln(K_D_) vs. 1/T for adsorption of MB on PVPA-BMEP hydrogels.

The thermodynamic parameters, including K_D_, ΔH°, and ΔS°, and ΔG°, were computed by applying Equations (7) and (8) to the isotherm data obtained at temperatures 299, 308, 318, and 328 K (Table 4). Accordingly, the thermodynamic parameters were evaluated in order to reveal the impact of temperature on the MB adsorption onto hydrogels. The plots of ln(K_D_) against 1/T in Figure 9 resulted linear lines with R^2^ values of 0.991, 0.990, 0.989, and 0.989 for PVPA-BMEP (5%), PVPA-BMEP (10%), PVPA-BMEP (20%), and PVPA-BMEP (40%) hydrogels, respectively. The negative ΔG° values of all hydrogels imply a feasible and spontaneous MB adsorption process. The ΔG° value of each hydrogel increases against increasing temperature from 299 to 328 K, corroborating that q_e_ values of hydrogels reduce at elevated temperature. Temperature change from 299 to 328 K induces 30.7%, 15.2%, 10.9%, and 8.6% relative reduction in q_e_ values for PVPA-BMEP (5%), PVPA-BMEP (10%), PVPA-BMEP (20%), and PVPA-BMEP (40%) hydrogels, respectively. This result suggests that the hydrogels with higher BMEP fractions (20% and 40%) are more effective on MB adsorption at ambient temperature conditions. The MB adsorption process on hydrogels is exothermic in nature since ΔH° values were found to be negative. As shown in Table 4, the negative ΔS° values signify the reduction in the randomness of solid/liquid interface through the adsorption process, which could be linked to the homogeneous adsorption mechanism on the surface of hydrogels previously inferred in Sections “3.3. Adsorption kinetics” and “3.4. Adsorption isotherms”. The results concluded here are similar to those reported in the literature for chitosan-g-poly(acrylic acid) hydrogels [52], calcium alginate-bentonite-activated carbon composite beads [23], bentonite-layered double hydroxide composite [73], starch-NiFe-layered double hydroxide composites [76], and monolithic graphene oxide gels [77].

### 3.6. Literature Comparison

The maximum MB adsorption capacities (q_m_) of hydrogels obtained in this study were compared to those reported in the literature for phosphonic acid-based adsorbents, environmentally friendly adsorbents, and highly efficient adsorbents with different compositions and structures (Table 5). The adsorption isotherms and the adsorption kinetics of the studies listed in Table 5 were described well by Langmuir and PSO models, respectively. The electrostatic attraction was reported to be the main mechanism for the adsorption of MB on the adsorbents shown in Table 5. The adsorption capacities of the adsorbents in Table 5 reached their maximum values in the MB solutions at neutral pH. The q_m_ values acquired in this study are much higher than those of phosphonic acid-based adsorbents and environmentally friendly adsorbents, demonstrating q_m_ values within the range of 14–524 mg g^−1^ [17,48,49,50,51,78,79,80]. The q_m_ values of hydrogels prepared in this study are also higher than those of adsorbents reported as highly efficient: chitosan-g-poly(acrylic acid) hydrogels improved with cellulose nanowhiskers (q_m_ = 2074 mg g^−1^) [52] and sodium alginate-based organic/inorganic superabsorbent composite hydrogels (q_m_ = 2257 mg g^−1^) [71]. The q_m_ value of the sodium alginate-based cross-linked beads reported by Shao, et al. [81] is 4.6% higher than that of PVPA-BMEP (40%) hydrogel. However, the contact time to reach the q_m_ value of 2977 mg g^−1^ by those beads is 24 times longer than that needed for the hydrogels of this study. Such longer contact times usually requires more number of tanks or larger tanks in case of application in continuous flow wastewater treatment plants with high flow rates, which may not be economically feasible for MB treatment at an industrial scale. This comparison suggests that the hydrogels produced in this study can be used as an effective adsorbent for MB treatment in wastewaters due to their outstanding adsorption capacities and rapid adsorption contact time compared to those reported in aforementioned studies.

The cost estimation for wastewater treatment is an important factor regarding the economic feasibility and sustainability of the proposed treatment technology. The water treatment technologies such as ion exchange, reverse osmosis, electrolysis, and electro-dialysis costs between 10 and 500 US$ per m^3^ of treated water. The water treatment cost by using adsorption technologies varies between 5 and 200 US$ per m^3^ of treated water [82]. On the other hand, it was reported that the cost of naturally available activated carbon-based adsorbents falls within the range between 0.02 and 20 US$ kg^−1^ [18,83]. The costs of commercially available activated carbon and the Filtrasorb 400 activated carbon were specified as 2 and 20 US$ kg^−1^, respectively [1,83]. The MB-bearing wastewater treatment cost by using the produced hydrogels in this study is about 150 US$ per m^3^ of treated water. Even though the estimated cost of the produced hydrogels is within the range as mentioned above for adsorption technologies, it is higher than those of naturally and commercially available activated carbon -based adsorbents. The added value to the produced hydrogels is their superior adsorption capacity and faster adsorption contact time compared to the previously reported adsorbents used for MB treatment from wastewaters.

## 4. Conclusions

Our motivation to initiate this research was to design and fabricate unique polymer hydrogels with outstanding dye adsorption capacities and fast adsorbate/adsorbent contact time for the management of environmental colored pollutants arising from dye-bearing wastewaters. To achieve this, the crosslinker bis[2-(methacryloyloxy)ethyl] phosphate was used in the synthesis of PVPA hydrogels adsorbent via free-radical polymerization of VPA. The FT-IR, TGA, and SEM results revealed that increasing BMEP fraction in the crosslinked PVPA-BMEP hydrogels leads to improvements in: (i) functionalities and pathways of the phosphonic acidic functional groups in the structure of hydrogels, (ii) thermal stabilities of hydrogels up to 250 °C, and (iii) interaction between MB molecules and hydrogels. The initial solution pH was found to be a significant parameter acting on the MB adsorption which was maximized at the neutral pH band, implying that the more available negatively charged functional groups on the surface of the hydrogels enhanced the electrostatic attraction between MB molecules and hydrogels. The experimental data obtained from batch adsorption experiments for MB removal fit best to the PSO kinetic model. The results of isotherm models implied that the behaviors of the isotherms are more appropriate for the Langmuir than the Freundlich isotherm for the experimental data. The highest monolayer adsorption capacity was found to be 2841 mg g^−1^ for the PVPA-BMEP (40%) hydrogel since it has more abundant phosphonic acidic functional groups in its structure. The thermodynamic studies indicated that the MB adsorption process on the hydrogels is spontaneous and exothermic in nature. The PVPA-BMEP (40%) hydrogel was found to be superior to the previously reported phosphonic acid-based adsorbents and highly efficient adsorbents with different compositions due to its outstanding adsorption capacity and fast adsorption equilibrium time. All these results rank the PVPA-BMEP hydrogels as a promising adsorbent for an efficient treatment of industrial dyes in wastewaters. The future work of this study is to enhance the produced adsorbent material regarding its cost, reusability, and biodegradability.

## Figures and Tables

**Table 1:** The kinetic models, their linear forms, and plot types to calculate the model parameters.

Kinetic Model	Linear Form	Equation No	Plot
Pseudo first-order	$\ln\left(q_{e}-q_{t}\right)=\ln\left(q_{e}\right)-k_{1}t$	(a)	ln(q_e_ − q_t_) vs. t
Pseudo second-order	$\frac{t}{q_{t}}=\frac{1}{k_{2}\;{q_{e}}^{2}}\;+\;\frac{t}{q_{e}}$	(b)	$\frac{t}{q_{t}}\;\mathrm{vs}.\;t$
Intraparticle diffusion	$q_{t}=k_{d}\;t^{0.5}\;+\;C$	(c)	q_t_ vs. t^0.5^
Elovich	$q_{t}=\frac{1}{\beta }\ln\left(\alpha \beta \right)\;+\;\frac{1}{\beta }\ln\left(t\right)$	(d)	q_t_ vs. ln(t)

**Table 2:** Parameters of kinetic models for MB adsorption on PVPA-BMEP hydrogels.

Adsorbent	C_0_	q_e,exp_	Pseudo First-Order			Pseudo Second-Order			Intraparticle Diffusion			Elovich		
			q_e,cal_	k_1_	R^2^	q_e,cal_	k_2_ × 10^−3^	R^2^	k_d_	C	R^2^	α	β × 10^−3^	R^2^
PVPA-BMEP (5%)	100	184	16.3	0.055	0.821	185	5.72	0.999	1.48	168	0.607	1.39 × 10^12^	168	0.758
	500	899	101	0.043	0.852	909	0.877	0.999	8.90	799	0.667	2.73 × 10^10^	28.0	0.822
	1500	2562	1407	0.059	0.990	2632	0.102	0.999	56.2	1915	0.804	2.27 × 10^5^	4.59	0.924
PVPA-BMEP (10%)	100	193	14.4	0.048	0.793	192	5.63	0.999	1.66	175	0.552	1.10 × 10^11^	146	0.723
	500	934	118	0.045	0.853	943	0.764	0.999	10.1	820	0.675	3.65 × 10^9^	24.7	0.830
	1500	2673	792	0.052	0.844	2703	0.114	0.999	56.1	2036	0.751	3.66 × 10^5^	4.56	0.878
PVPA-BMEP (20%)	100	197	21.3	0.035	0.797	200	3.29	0.999	2.49	169	0.570	2.04 × 10^7^	97.5	0.741
	500	958	252	0.053	0.882	980	0.325	0.999	23.4	702	0.523	1.75 × 10^4^	10.2	0.699
	1500	2766	2405	0.058	0.971	2941	0.034	0.998	131	1285	0.724	9.24 × 10^2^	1.92	0.871
PVPA-BMEP (40%)	100	197	42.4	0.060	0.878	200	2.03	0.999	3.90	154	0.543	3.10 × 10^4^	62.0	0.711
	500	966	282	0.058	0.750	1010	0.162	0.997	36.4	568	0.529	8.83 × 10^2^	6.60	0.702
	1500	2805	2753	0.063	0.869	3226	0.016	0.982	177	827	0.688	3.35 × 10^2^	1.41	0.847

**Table 3:** Parameters, Δq_e_, and R^2^ values of the isotherm models for MB adsorption on PVPA-BMEP hydrogels.

Adsorbent	Langmuir				Freundlich				Redlich-Peterson				
	q_m_	k_L_ × 10^−3^	Δq_e_	R^2^	k_F_	1/n	Δq_e_	R^2^	k_rp_	α_rp_ × 10^−3^	β	Δq_e_	R^2^
PVPA-BMEP (5%)	2593	5.24	2.66	0.998	40.4	0.769	9.66	0.992	22.7	5.91	0.966	2.33	0.999
PVPA-BMEP (10%)	2724	11.1	3.15	0.997	76.0	0.721	11.3	0.989	45.2	10.3	0.980	3.31	0.992
PVPA-BMEP (20%)	2787	16.0	1.85	0.999	98.0	0.716	15.2	0.981	65.6	24.5	0.906	2.50	0.997
PVPA-BMEP (40%)	2841	19.2	2.45	0.998	116	0.710	12.3	0.987	85.9	35.4	0.888	0.32	0.999

**Table 4:** Thermodynamic parameters of MB adsorption on PVPA-BMEP hydrogels.

Adsorbent	T (K)	ΔG° (kJ moL^−1^)	ΔH° (kJ mL^−1^)	ΔS° (J mL^−1^ K^−1^)
PVPA-BMEP (5%)	299	−7.42	−46.9	−132
	308	−6.23		
	318	−4.91		
	328	−3.59		
PVPA-BMEP (10%)	299	−9.12	−41.6	−109
	308	−8.14		
	318	−7.06		
	328	−5.97		
PVPA-BMEP (20%)	299	−9.94	−40.3	−102
	308	−9.03		
	318	−8.01		
	328	−6.99		
PVPA-BMEP (40%)	299	−10.5	−39.6	−97.3
	308	−9.67		
	318	−8.69		
	328	−7.72		

**Table 5:** Comparison of MB adsorption capacities of different adsorbents.

Adsorbent	q_m_ (mg g^−1^)	C_0_ (mg L^−1^)	t_e_ (min)	Reference
PVPA nanogels	14	30	5	[49]
Brown macroalga (N. zanardinii)	35	200	120	[80]
Activated lignin-chitosan extruded blends	36	80	180	[17]
Poly(acrylic acid-co-VPA) hydrogel cross-linked with N-maleyl chitosan	67	50	100	[50]
Chemically modified pine nut shells in single and binary systems	137	200	90	[79]
Arginine modified activated carbon	220	250	120	[78]
PVPA-co-triethyleneglycol dimethacrylate	417	2100	360	[51]
Phosphonic acid functionalized benzene-bridged periodic mesoporous organosilicas	524	600	320	[48]
Chitosan-g-poly(acrylic acid) hydrogels improved with cellulose nanowhiskers	2074	2000	60	[52]
Sodium alginate-based organic/inorganic superabsorbent composite hydrogel	2257	600	400	[71]
Sodium alginate-based cross-linked beads	2977	160	1500	[81]
PVPA-BMEP (5%)	2593	1500	60	This study
PVPA-BMEP (10%)	2724			
PVPA-BMEP (20%)	2787			
PVPA-BMEP (40%)	2841			

## Supplementary Materials

The following are available online at https://www.mdpi.com/2079-4991/10/1/131/s1, Figure S1: Zeta potential and ΔpH plots of PVPA-BMEP (40%) hydrogel at different pH_initial_ values., Figure S2: The change in R_L_ value of each hydrogel within the studied C_0_ range., Table S1: Parameters and R^2^ values of two-step linear plots of IPD model.
