# Supplementary material for: Design of Crosslinked Hydrogels Comprising Poly(Vinylphosphonic Acid) and Bis[2-(Methacryloyloxy)Ethyl] Phosphate as an Efficient Adsorbent for Wastewater Dye Removal
Source: Nanomaterials (Basel). 2020 Jan 10;10(1):131. doi: 10.3390/nano10010131 (PMC7023366; doi:10.3390/nano10010131)
Supplement: Supplementary file 1 [file nanomaterials-10-00131-s001.pdf]

Supplementary Material

List OF Supplementary Tables

Table S1. Parameters and R^2 values of two-step linear plots of IPD model.

Adsorbent	C_0	1st Step			2nd Step		
		k_{d1}	C_1	R^2	k_{d2}	C_2	R^2
PVPA-BMEP (5%)	100	6.29	146	0.893	0.017	184	0.994
	500	34.5	683	0.972	0.351	894	0.994
	1500	148	1490	0.983	11.0	2424	0.823
PVPA-BMEP (10%)	100	8.66	144	0.996	0.034	192	0.994
	500	38.5	692	0.981	0.351	929	0.994
	1500	147	1603	0.937	1.05	2659	0.994
PVPA-BMEP (20%)	100	12.7	124	0.999	0.198	194	0.994
	500	128	239	0.973	0.499	951	0.972
	1500	428	−78.5	0.994	5.99	2690	0.872
PVPA-BMEP (40%)	100	20.6	79.8	0.979	0.143	195	0.854
	500	199	−150	0.995	0.204	963	0.924
	1500	639	−1,276	0.995	1.60	2784	0.987

List of Supplementary Figures

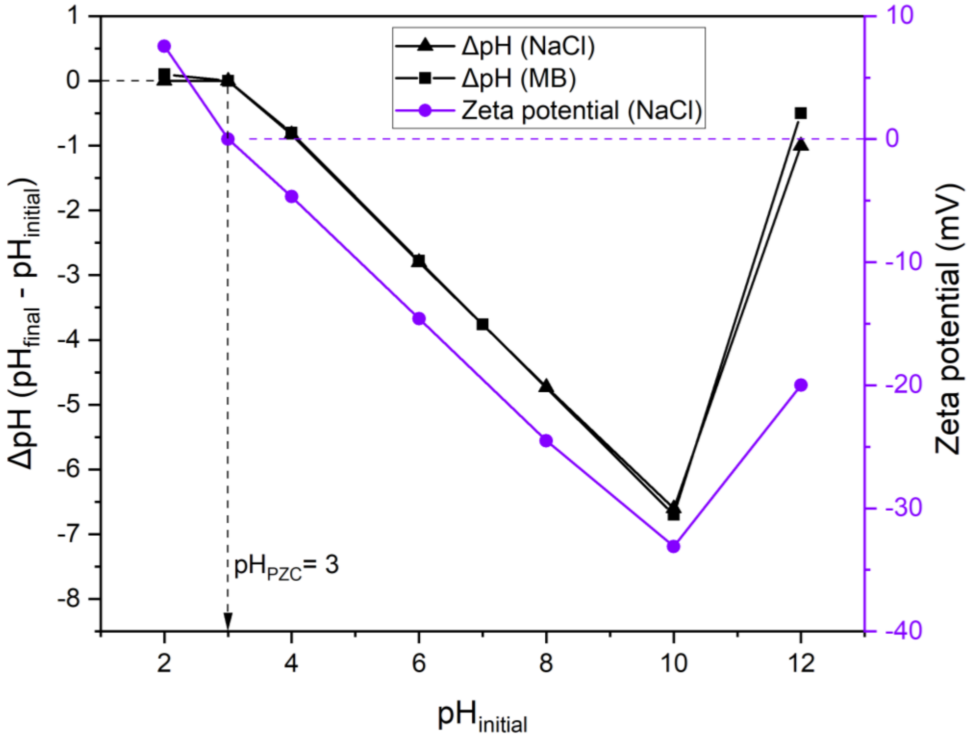

Figure S1. Zeta potential and ΔpH plots of PVPA-BMEP (40%) hydrogel at different $pH_{initial}$ values.

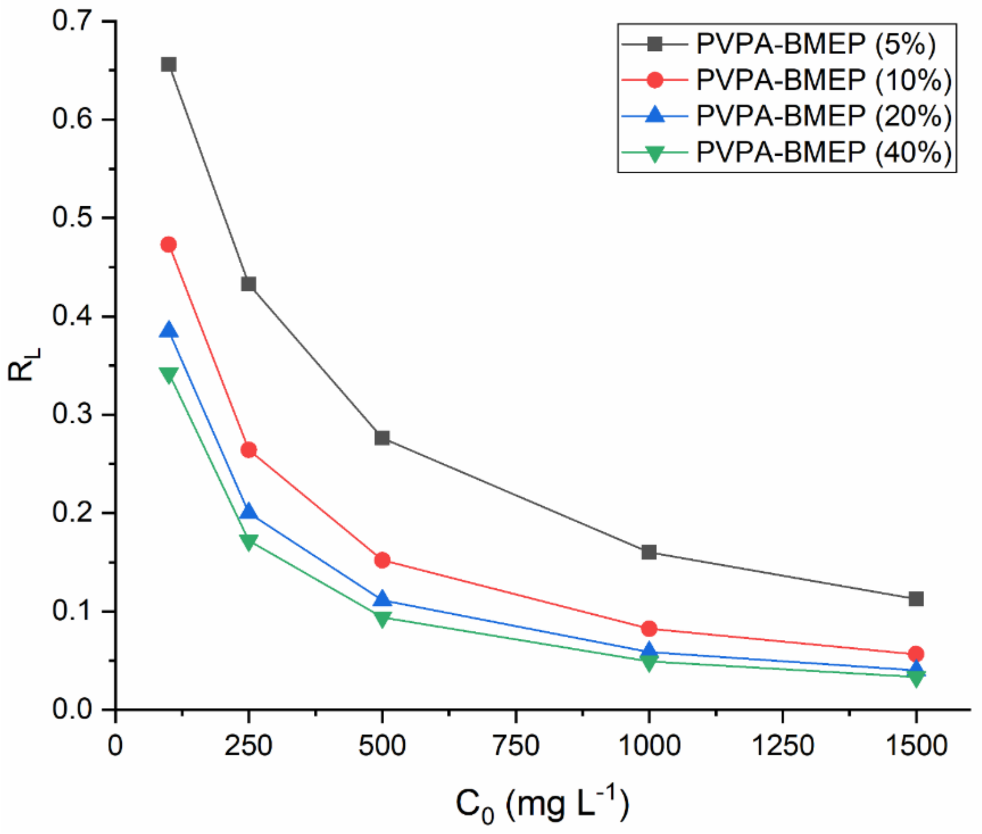

Figure S2. The change in R_L value of each hydrogel within the studied C_0 range.
